# Crosstalk between PI3K/AKT signaling and epigenetic regulation in hepatocellular carcinoma: molecular mechanisms and therapeutic opportunities

**DOI:** 10.3389/fcell.2026.1975140

**Published:** 2026-10-20

**Authors:** Zhibo Zhang, Jie Liu, Guofeng Lei, Xinjun Hu

**Affiliations:** 1 The First Affiliated Hospital, College of Clinical Medicine, Henan University of Science and Technology, Luoyang, Henan, China; 2 The First Affiliated Hospital of Zhengzhou University, Zhengzhou, Henan, China; 3 Yellow River Sanmenxia Hospital Affiliated to Henan University of Science and Technology, Sanmenxia, Henan, China

**Keywords:** epigenetic regulation, hepatocellular carcinoma, molecular mechanisms, PI3K/akt signaling pathway, targeted therapy

## Abstract

Hepatocellular carcinoma (HCC) is an aggressive malignancy, and most patients are diagnosed at intermediate or advanced stages. Targeted therapies, immunotherapies, and combination regimens have limited efficacy, creating a major clinical challenge. The phosphatidylinositol 3-kinase/protein kinase B (PI3K/AKT) pathway is frequently and persistently activated in HCC. Epigenetic modifications regulate gene expression without altering the DNA sequence and contribute to malignant progression. Aberrant epigenetic states sustain PI3K/AKT activation by silencing tumor-suppressor genes and activating oncogenic signaling. Conversely, activated PI3K/AKT signaling remodels the epigenome through several enzymes that mediate epigenetic modifications. This bidirectional interaction promotes HCC progression. This review examines how DNA methylation, non-coding RNAs, and histone modifications drive excessive PI3K/AKT pathway activation. It also assesses the more limited evidence that PI3K/AKT signaling regulates these enzymes and reviews recent progress in combining PI3K inhibitors with epigenetic-targeting agents. We then consider the main barriers to clinical translation, including biomarker selection, treatment scheduling, and nanocarrier-based co-delivery. Clarifying this reciprocal regulation may support more rational, individualized therapeutic strategies for HCC.

## 1 Introduction

Hepatocellular carcinoma (HCC) is a common malignancy of the digestive system characterized by an aggressive course and poor long-term survival (Chen et al., 2026; Sanchez-Martin et al., 2023). Because early HCC often lacks specific symptoms, many patients are ineligible for curative resection at diagnosis (Zhou and Song, 2021). Multiple treatment options are available for advanced HCC, but overall survival remains limited and therapeutic responses are often unsatisfactory (Yau et al., 2025; Sangro et al., 2024; Rimassa et al., 2025). A clearer understanding of the molecular pathogenesis of HCC is therefore needed to address these clinical challenges and refine treatment strategies (Wang S. et al., 2025).

Among the signaling cascades implicated in HCC progression, the PI3K/AKT pathway regulates hepatocyte proliferation, apoptosis, and metabolism (Paskeh et al., 2023). Persistent pathway activation promotes HCC cell proliferation, invasion, and metastasis through downstream effectors, thereby contributing to HCC initiation and progression (Guo et al., 2024; Sun et al., 2024). In addition to genetic alterations, epigenetic modifications influence HCC without changing the DNA sequence (Bueloni et al., 2025). These mechanisms include DNA methylation, histone modification, and chromatin remodeling. Non-coding RNAs primarily regulate gene expression post-transcriptionally, although some long non-coding RNAs (lncRNAs) can also modulate chromatin. Dysregulation of these mechanisms can silence tumor-suppressor genes and activate oncogenes, promoting malignant transformation and hepatocarcinogenesis (Fernandez-Barrena et al., 2025; Nagaraju et al., 2022).

Recent studies indicate that activated PI3K/AKT signaling can regulate the expression and catalytic activity of epigenetic modifiers, thereby reshaping the epigenetic state of HCC cells (Yang et al., 2019; Su X. et al., 2024). Conversely, aberrant epigenetic modifications can alter PI3K/AKT pathway activity (Liu et al., 2023a; Spangle et al., 2017). These reciprocal effects may jointly promote HCC initiation and progression (Castel et al., 2025; Wu et al., 2020). However, the available evidence remains fragmented. The effects of chronic liver disease, etiological heterogeneity, and the tumor microenvironment on this regulatory network have not been fully elucidated. In addition, the clinical implications of these mechanisms for biomarker-guided patient selection and treatment optimization remain unclear. This review therefore summarizes the PI3K/AKT-epigenetic regulatory network in HCC, evaluates its therapeutic relevance and current limitations, and identifies key priorities for future research on individualized treatment.

## 2 Overview of the PI3K/AKT signaling pathway

The evolutionarily conserved PI3K/AKT pathway regulates cell survival, growth, proliferation, apoptosis, and metabolic homeostasis. Its principal components include PI3K, AKT, PTEN, and multiple upstream and downstream regulators (Glaviano et al., 2023). Physiological pathway activity maintains cellular homeostasis, whereas dysregulation can contribute to malignant transformation (Tian et al., 2023). Among class I PI3Ks, class IA PI3K is most closely associated with malignant tumor phenotypes. It is a heterodimer comprising a p110 catalytic subunit and a p85 regulatory subunit (Li H. et al., 2024). Of the three AKT isoforms, AKT1 and AKT2 predominate in the liver and are the main functional isoforms in hepatocytes (Wang Z. et al., 2025). PTEN is a key negative regulator that restrains pathway activity (Qian et al., 2025). Extracellular growth factors activate PI3K through receptor tyrosine kinases at the cell surface. Ligand binding triggers receptor tyrosine kinase (RTK) dimerization and autophosphorylation, generating phosphotyrosine docking sites for class I PI3K recruitment and activation. The p85 regulatory subunit recognizes these phosphorylated tyrosine residues through its SH2 domains, thereby recruiting and activating the p110 catalytic subunit at the plasma membrane. Activated PI3K then converts phosphatidylinositol 4,5-bisphosphate (PIP2) to phosphatidylinositol 3,4,5-trisphosphate (PIP3) (Vivanco and Sawyers, 2002; Chu et al., 2026; Duewell et al., 2024). PIP3 promotes the recruitment of AKT and PDK1 to the plasma membrane through their pleckstrin homology domains. AKT is fully activated through phosphorylation by PDK1 at Thr308 and mTORC2 at Ser473 (Bang et al., 2023). Under physiological conditions, PTEN dephosphorylates PIP3, thereby limiting signal transduction and preventing excessive pathway activation (Li X. et al., 2024). Persistent PI3K/AKT activation is common across cancers and is particularly relevant to HCC (Zhao J. et al., 2025; Fontana et al., 2024). In HCC cells, this altered signaling promotes proliferation, invasion, and metastasis through downstream effectors (Zheng et al., 2025).

## 3 Overview of epigenetic regulation

Epigenetic regulation alters gene transcription without changing the genomic DNA sequence (Meza-Menchaca et al., 2024; Goldberg et al., 2007). It controls gene activation or silencing by modifying cis-regulatory elements, including promoters and enhancers, and by influencing transcription factor activity. Unlike DNA sequence mutations, epigenetic changes are dynamic and potentially reversible (Iyer et al., 2026; Hogg et al., 2020). Four major mechanisms are relevant here. In normal mammalian genomes, dispersed CpG sites are widely methylated, whereas promoter CpG islands generally remain hypomethylated to permit transcription. Tumor cells often acquire promoter CpG-island hypermethylation, which can silence tumor-suppressor genes (Lavoro et al., 2025; Lu et al., 2023; Nishiyama and Nakanishi, 2021). DNA methyltransferases (DNMTs) catalyze methylation, with DNMT1 primarily maintaining established methylation patterns. By contrast, ten-eleven translocation 1 (TET1) supports active demethylation through stepwise oxidation (Marei, 2025; Tahiliani et al., 2009; Davletgildeeva and Kuznetsov, 2024). Histone modifications occur on the exposed N-terminal tails of core histones and include acetylation, methylation, phosphorylation, and ubiquitination. Antagonistic enzyme systems dynamically regulate each modification (Zhang et al., 2021; Zaib et al., 2022; Liu R. et al., 2023). Histone acetyltransferases and histone deacetylases control acetylation, whereas methyltransferases and demethylases regulate methylation. Kinases and phosphatases regulate phosphorylation, and ubiquitin ligases and deubiquitinases regulate ubiquitination (Sadia et al., 2025; Millán et al., 2022). ATP-dependent chromatin remodeling, by contrast, changes nucleosome positioning without adding or removing chemical groups. SWI/SNF and ISWI complexes hydrolyze ATP to reposition or evict nucleosomes and alter chromatin accessibility. BRG1 is a catalytic ATPase within SWI/SNF complexes (Malone and Roberts, 2024; Barisic et al., 2019). Non-coding RNAs, including microRNAs (miRNAs), lncRNAs, and circular RNAs (circRNAs), provide another layer of regulation (Das et al., 2026; Nemeth et al., 2024; Yan and Bu, 2021). They can recruit epigenetic enzymes through sequence-specific interactions and influence post-transcriptional processes, including RNA m^6^A methylation and alternative splicing (Figure 1; Shi et al., 2025; Wu et al., 2026).

**FIGURE 1:**
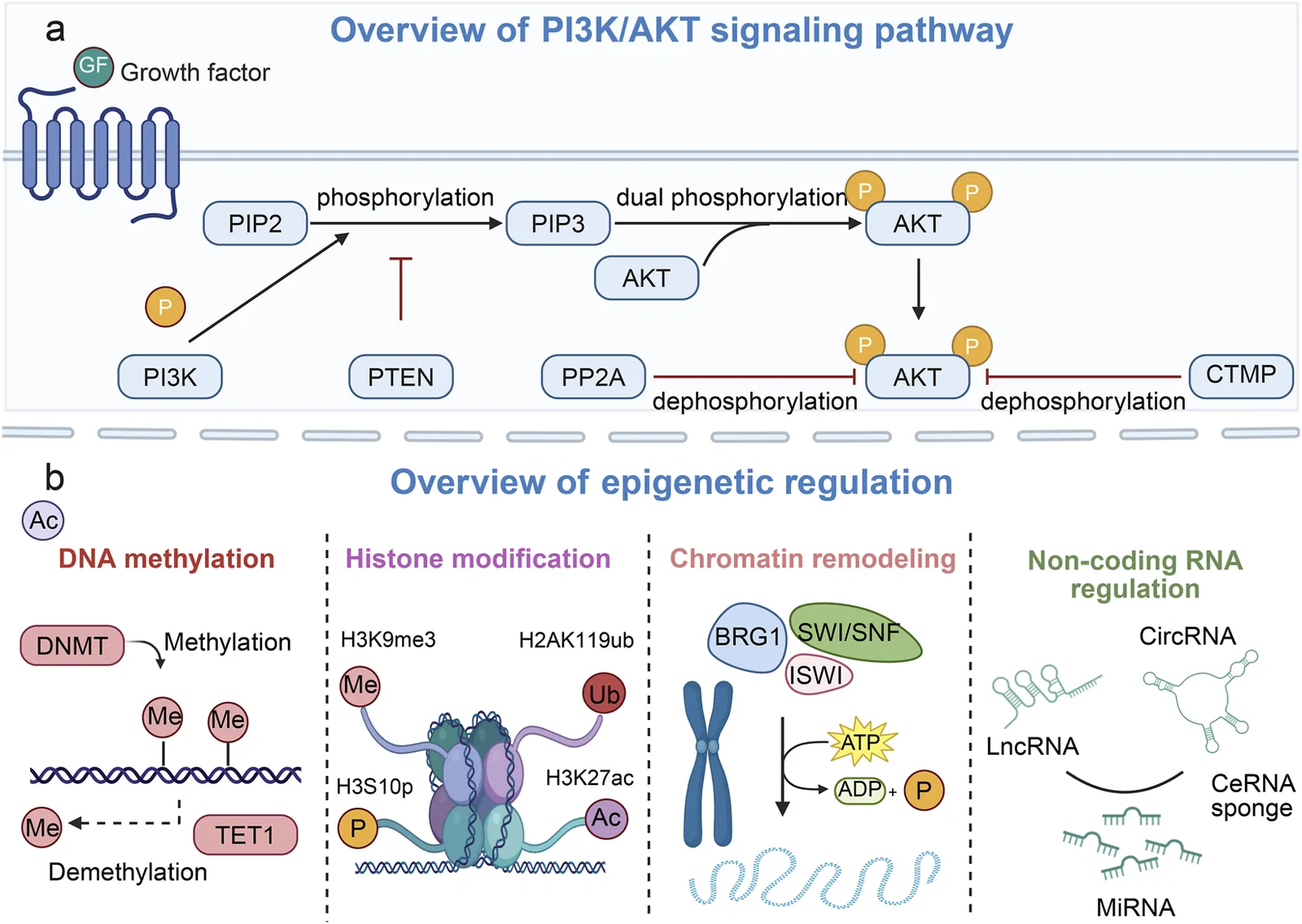
PI3K/AKT signaling and major epigenetic regulatory mechanisms. **(a)** The upper panel shows the phosphorylation and dephosphorylation events that control AKT activation. **(b)** The lower panel summarizes DNA methylation, histone modification, chromatin remodeling, and non-coding RNA-mediated regulation. Figure created with BioRender.com. GF, growth factor; PIP2, phosphatidylinositol 4,5-bisphosphate; PIP3, phosphatidylinositol 3,4,5-trisphosphate; PTEN, phosphatase and tensin homolog; PP2A, protein phosphatase 2A; CTMP, carboxyl-terminal modulator protein; DNMT, DNA methyltransferase; TET1, tet methylcytosine dioxygenase 1; BRG1, Brahma-related gene 1; SWI/SNF, switch/sucrose non-fermentable complex; ISWI, imitation switch complex; circRNA, circular RNA; lncRNA, long non-coding RNA; miRNA, microRNA; ceRNA, competing endogenous RNA; p, phosphorylation; me, methylation; ac, acetylation; ub, ubiquitination.

## 4 Epigenetic and RNA-mediated regulation of PI3K/AKT signaling in HCC

Evidence increasingly supports epigenetic alterations as upstream regulators of PI3K/AKT signaling in HCC. Aberrant DNA methylation, histone modifications, and non-coding RNA expression can sustain pathway activation. These changes are associated with HCC cell proliferation, epithelial-mesenchymal transition (EMT), metastasis, and therapeutic resistance. In HCC, these regulatory events are shaped by the underlying hepatic disease environment. This includes chronic liver injury, cirrhosis, persistent inflammation, metabolic dysregulation, hypoxia, and immune-stromal remodeling. Interactions among hepatocytes, hepatic stellate cells, endothelial cells, tumor-associated macrophages, and cancer-associated fibroblasts may further shape the activity of the PI3K/AKT signaling network (Matsuda and Seki, 2020; Barry et al., 2020; Zulaziz et al., 2023). In this context, hepatic metabolic signals may link hepatic metabolic status and microenvironmental stress to PI3K/AKT pathway activity. These signals involve the insulin/growth factor-PI3K/AKT/mTOR axis, hypoxia-induced non-coding RNA (ncRNA) regulation, enhanced glycolysis, and lactate-associated histone lactylation (Tian et al., 2023; Hu et al., 2017; Zeng et al., 2025). Paracrine signals mediated by macrophages and fibroblasts may also connect the immune-stromal microenvironment with PI3K/AKT pathway activity and therapeutic responses (Xue et al., 2026; Wan et al., 2026). These context-dependent interactions may contribute to the biological heterogeneity of HCC and influence therapeutic responses (Table 1).

**TABLE 1:** Mechanisms linking PI3K/AKT signaling and epigenetic regulation in HCC.

Cancer type	Epigenetic mechanism	Related molecules	Mechanism	Cell lines	References
HCC	DNA methylation	DNMT1, ZNF191	ZNF191 binds the DNMT1 promoter → DNMT1 transcription and expression increase → DNA methylation is remodeled → PI3K/AKT signaling is activated → HCC cell proliferation increases	Hep3B, PLC/PRF/5, SK-Hep-1, L02, HEK-293T	Liu et al. (2022)
HCC	DNA methylation	DNMT1, Six2	Six2 overexpression → DNMT1 upregulation → CpG hypermethylation of the E-cadherin promoter → E-cadherin repression and PI3K/AKT/mTOR activation → increased HCC cell proliferation, invasion, and 5-FU resistance	Huh7, Hep3B	Li et al. (2024c)
HCC	DNA methylation	DNMT3B, TERT, Sp1	TERT cooperates with Sp1 → DNMT3B upregulation → PTEN and RASSF1A promoter hypermethylation → PTEN and RASSF1A silencing → sustained AKT activation → accelerated HCC progression	PLC/PRF/5, HUH-7	Yu et al. (2018)
HCC	DNA methylation (hypermethylation)	DNMT1, DNMT3A, DNMT3B, NNAT	DNMT1/DNMT3A/DNMT3B ↑ → *NNAT* promoter hypermethylation → *NNAT* silencing → PI3K/AKT pathway activation → HCC cell proliferation and progression	Hep3B, SK-Hep1	Deng et al. (2023)
HCC	DNA methylation (hypermethylation)	DNMT1, DNMT3A, DNMT3B, PDE7B	DNA methyltransferases ↑ → PDE7B promoter CpG hypermethylation → PDE7B downregulation → sustained PI3K/AKT activation → EMT and increased HCC cell proliferation and migration	L02, SMMC-7721, MHCC-97H, Huh7, SK-Hep-1	Du et al. (2024)
HCC	DNA methylation (hypomethylation)	FBXO32	FBXO32 promoter hypomethylation → FBXO32 upregulation → PHLPP2 binding and ubiquitin–proteasome-mediated degradation → PI3K/AKT activation → HCC progression	THLE-2, HLF, Huh7, Hep3B, MHCC97L, MHCC97H, HEK293T	Zeng et al. (2025)
HCC	DNA demethylation	TET1	TET1 demethylase activity → PI3K/AKT/mTOR activation → proliferation ↑/apoptosis ↓	Huh-7, HepG2, Hep3B, LO2	Qi et al. (2026)
HCC	Long non-coding RNA regulation	lncRNA IGFRIL, PTBP1	lncRNA IGFRIL ↑ → recruits PTBP1 → induces IGFBP3 mRNA degradation → IGFBP3 ↓ → hyperactivation of the IGF1R-AKT-mTOR pathway → enhanced malignant HCC phenotypes	HepG2, Huh7, JHH2, MHCC97H, HEK293 T	Zhang et al. (2025a)
HCC	Long non-coding RNA regulation	lncRNA USP27X-AS1, SP1, USP7	SP1 ↑ → USP27X-AS1 ↑ → USP7 recruitment → reduced AKT ubiquitination and increased AKT stability → persistent PI3K/AKT activation → HCC cell proliferation and metastasis	HLF, Hep3B, LM3, MHCC97H, HepG2, THLE3, HEK293 T	Su et al. (2024c)
HCC	Long non-coding RNA regulation	HIF-1α, lncRNA KDM4A-AS1, miR-411–5p	HIF-1α ↑ → lncRNA KDM4A-AS1 ↑ → sequesters miR-411–5p → KPNA2 ↑ → AKT pathway activation → promotes HCC cell proliferation and metastasis; meanwhile, KPNA2/AKT ↑ → HIF-1α ↑, forming a positive feedback loop	Hep3B, Huh7, HepG2, MHCC-97H, HCCLM3, SK-Hep-1, MIHA, HEK293 T	Chen et al. (2021)
HCC	Long non-coding RNA regulation	E2F1, lncRNA KDM4A-AS1, ILF3	E2F1 ↑ → binds to the KDM4A-AS1 promoter and activates transcription → lncRNA KDM4A-AS1 ↑ → recruits ILF3 → increased AURKA mRNA stability and AURKA upregulation → activation of the PI3K/AKT pathway → proliferation, invasion, and EMT of HCC cells	Hep3B, HCCLM3, MHCC97-L, Huh-7, THLE-3, HEK293 T	Shen et al. (2023)
HCC	Long non-coding RNA regulation	HIF-1α, lncRNA MAPKAPK5-AS1, miR-154–5p	Hypoxia → HIF-1α ↑ → lncRNA MAPKAPK5-AS1 ↑ → miR-154–5p sequestration → PLAGL2 ↑ → EGFR/PI3K/AKT activation and HCC cell proliferation and invasion; PLAGL2 also increases HIF-1α, forming a positive feedback loop	Huh7, Hep3B, MHCC97-H, MHCC97-L, HepG2, HCCLM3, L02, HEK293 T	Wang et al. (2021)
HCC	Long non-coding RNA regulation	lncRNA RP11-295G20.2	RP11-295G20.2 ↑ → directly binds PTEN → promotes PTEN lysosomal degradation → loss of PTEN-mediated AKT inhibition → PI3K/AKT hyperactivation → autophagy suppression → HCC progression	Huh7, SK-Hep1, MHCCLM3, HepG2, HEK293 T	Liang et al. (2021)
HCC	Circular RNA regulation	circMRPS35, miR-148a	circMRPS35 ↑ → miR-148a sponging → STX3 upregulation → increased PTEN ubiquitination and degradation → sustained PI3K/AKT activation → HCC cell proliferation and metastasis	Huh-7, HCC-LM3, HepG2, SNU-398, SMMC-7721, L02, 293 T	Li et al. (2022)
HCC	Circular RNA regulation	circACVR2A, miR-511–5p	circACVR2A ↑ → miR-511–5p sponging and inhibition → PI3K/AKT activation → HCC progression	Huh-7, HepG2, Hep3B, LO2, MIHA, HEK293 T	Fei et al. (2024)
HCC	Circular RNA regulation	circZKSCAN1, circZKSaa	circZKSCAN1 ↑ → circZKSaa translation → increased FBXW7-mTOR binding → mTOR ubiquitination and degradation → PI3K/AKT/mTOR inhibition → reduced HCC progression and increased sorafenib sensitivity	HCC-LM3, SNU-398, Hep3B, Huh-7, SNU-387, THL-2, 293T, Hela	Song et al. (2023)
HCC	Histone methylation	SUV39H1, H3K9me3, SAM	PCK1 ↓ → SAM synthesis ↓ → SUV39H1 methyltransferase activity impaired → repressive H3K9me3 modification ↓ at the S100A11 promoter → S100A11 transcriptional derepression and upregulation → S100A11 binds and activates AKT1 → sustained PI3K/AKT pathway activation → HCC cell proliferation, invasion, and metastasis	PLC/PRF/5, SNU449, SK-Hep1, MHCC-97H, Hep1-6, HEK293	Gou et al. (2023)
HCC	Histone methylation	EZH2, H3K27me3	EZH2 ↑ → catalyzes H3K27me3 at the miR-622 promoter → silences miR-622 → derepresses CXCR4 → CXCL12-CXCR4 axis → activates PI3K/AKT and RAF/MEK/ERK → drives malignant HCC progression	Huh7, SK-Hep1, HepG2, Hep3B, PLC/PRF/5, SNU448, HCC-LM3	Wang et al. (2023)
HCC	Histone acetylation	ACLY, H3K27ac, acetyl-CoA	Macrophage GOLM1 ↑ → binds and inhibits ACLY → nuclear P-ACLY ↓ → acetyl-CoA ↓ → H3K27ac modification at the EFEMP1 promoter ↓ → EFEMP1 transcription and secretion ↓ → EFEMP-EGFR binding reduced → PI3K/AKT and MAPK pathway suppression → HCC progression inhibited	THP1, BMDM, Huh7, HepG2, Hepa1-6, HEK293 T	Zhen et al. (2026)
HCC	Histone acetylation	MBD3, HDAC1, H3K27ac	MBD3 ↑ → recruits the NuRD complex to the TFPI2 promoter → histone deacetylation → TFPI2 transcriptional silencing → release of PI3K/AKT inhibition → sustained PI3K/AKT activation → HCC	Hep3B, SK-Hep1, HepG2, Huh7, HEK293 T	Yan et al. (2022)
HCC	Histone lactylation	PYCR1, H3K18La	PYCR1 ↑ → glycolysis enhanced → intracellular lactate ↑ → H3K18 lactylation enrichment at the IRS1 promoter → IRS1 transcriptional activation → PI3K/AKT/mTOR and MAPK/ERK signaling upregulation → HCC cell proliferation and metastasis	HepG2, HuH-7	Wang et al. (2024)
HCC	Histone lactylation	H3K14La, NEDD4	Glycolysis ↑ → lactate accumulation → H3K14La upregulation → NEDD4 transcription upregulated → PTEN ubiquitination and degradation → persistent PI3K/AKT/mTOR activation → HCC chemoresistance	LM3, Huh7	Zeng et al. (2025)
HCC	DNA methylation	DNMT3B	Activated AKT → elevates HuR expression → extends DNMT3B mRNA half-life/increases DNMT3B translation; meanwhile, AKT activates DNMT3B transcription → DNMT3B accumulation → disrupts global DNA methylation → promotes malignant HCC phenotypes	BEL-7404, BEL-7402, BEL-7405, QGY-7703, SMMC-7721, HepG2, L02	Mei et al. (2010)
HCC	Histone acetylation	p300, H3K9ac	EGF/EGFR activation → activates PI3K/AKT → AKT phosphorylates p300 at Ser 1834 → enhances p300 acetyltransferase activity → increases H3K9 acetylation at the HMGA2 promoter → upregulates HMGA2 transcription → promotes HCC cell invasion and metastasis	SNU-368, SNU-739, Huh-7, HepG2, H22	Liang et al. (2021)
HCC	Histone acetylation and histone phosphorylation	p300, H3K9ac, H3T11 ph, DKK1	EGF/EGFR → PI3K/AKT → p300 phosphorylation → H3K9 acetylation at the DKK1 promoter; EGF/EGFR → MEK/ERK → PKM2 phosphorylation and nuclear translocation → H3T11 phosphorylation at the DKK1 promoter → increased HCC cell invasion and metastasis	Huh-7, HepG2	Niu et al. (2020)
HCC	Histone deacetylation	HDAC2, Cdc25c, cyclin B	EGF stimulation → upregulates CK2α → promotes PI3K/AKT phosphorylation → elevates HDAC2 expression → upregulates Cdc25c and cyclin B → accelerates the G2/M transition → promotes malignant HCC cell proliferation	Huh-7, Hep3B, SNU-449	Kim et al. (2014)

In this review, DNA methylation and histone modifications are considered canonical epigenetic mechanisms. They regulate gene expression through changes in DNA methylation status, chromatin structure, or histone marks without altering the DNA sequence. ncRNA-mediated effects on mRNA stability, translation, protein ubiquitination, or protein degradation are discussed separately as RNA-mediated post-transcriptional or protein-level regulation. lncRNAs that recruit chromatin-modifying complexes may also participate in epigenetic regulation (Shi et al., 2025; Costa et al., 2025).

### 4.1 Effects of dysregulated DNA methylation on PI3K/AKT signaling

Altered activity of DNMTs and TET demethylases can disrupt PI3K/AKT pathway regulation and influence HCC cell proliferation, invasion, EMT, and chemoresistance (Cheng et al., 2024; Su L. et al., 2024). Mechanistically, dysregulated DNA methylation drives aberrant PI3K/AKT activation through three core mechanisms. DNMT upregulation induces widespread epigenetic repression, and tumor-suppressor gene silencing removes inhibitory constraints on the pathway. Hypomethylation-mediated upregulation of protumorigenic genes further enhances oncogenic signaling. DNMT1 is an important mediator of aberrant pathway activation. Zinc finger protein 191 (ZNF191) binds the DNMT1 promoter and increases DNMT1 transcription. DNMT1 overexpression alters genome-wide methylation patterns and is associated with PI3K/AKT pathway activation (Liu et al., 2022). The oncogenic activity of the transcription factor Six2 also involves DNMT1-dependent methylation. Six2 enhances DNMT1-mediated methylation of the E-cadherin promoter, reducing E-cadherin expression and promoting EMT. It also activates PI3K/AKT/mTOR signaling, accelerates HCC progression, and contributes to 5-fluorouracil (5-FU) resistance (Li J. et al., 2024). DNA methyltransferase 3B (DNMT3B) provides another route to excessive pathway activation. Telomerase reverse transcriptase (TERT) cooperates with transcription factor Sp1 to increase DNMT3B expression. This interaction promotes hypermethylation and silencing of tumor-suppressor genes, including PTEN and RASSF1A. Loss of PTEN-mediated inhibition sustains AKT activation and promotes malignant progression (Yu et al., 2018). Silencing these tumor suppressors removes endogenous constraints on AKT phosphorylation, thereby sustaining PI3K/AKT activation. DNMT family proteins also alter the methylation of other tumor-suppressor genes. Hypermethylation of the NNAT and PDE7B promoters reduces the expression of their encoded proteins. Because both proteins suppress phosphorylation of PI3K/AKT pathway kinases, their loss weakens this restraint. Reduced NNAT expression primarily promotes HCC cell proliferation, whereas reduced PDE7B expression promotes EMT (Deng et al., 2023; Du et al., 2024). Thus, DNMT upregulation can simultaneously silence multiple pathway-inhibitory genes, cumulatively favoring persistent PI3K/AKT signaling.

In addition to DNMT-mediated silencing of tumor-suppressor genes, hypomethylation of oncogene promoters can amplify PI3K/AKT signaling. Hypomethylation of the FBXO32 promoter increases FBXO32 expression. FBXO32 promotes ubiquitin–proteasome-mediated degradation of the tumor suppressor PHLPP2, thereby reducing PHLPP2-mediated inhibition of AKT and sustaining malignant phenotypes in HCC (Zeng et al., 2025).

Aberrant TET1-mediated demethylation can also disrupt PI3K/AKT pathway regulation. Through its demethylase activity, TET1 promotes PI3K/AKT/mTOR signaling, increases HCC cell proliferation, and suppresses apoptosis (Qi et al., 2026). Thus, promoter hypermethylation of tumor-suppressor genes, promoter hypomethylation of oncogenes, and aberrant TET1-mediated demethylation can converge on persistent PI3K/AKT activation. The available evidence links these mechanisms to several malignant HCC phenotypes (Figure 2).

**FIGURE 2:**
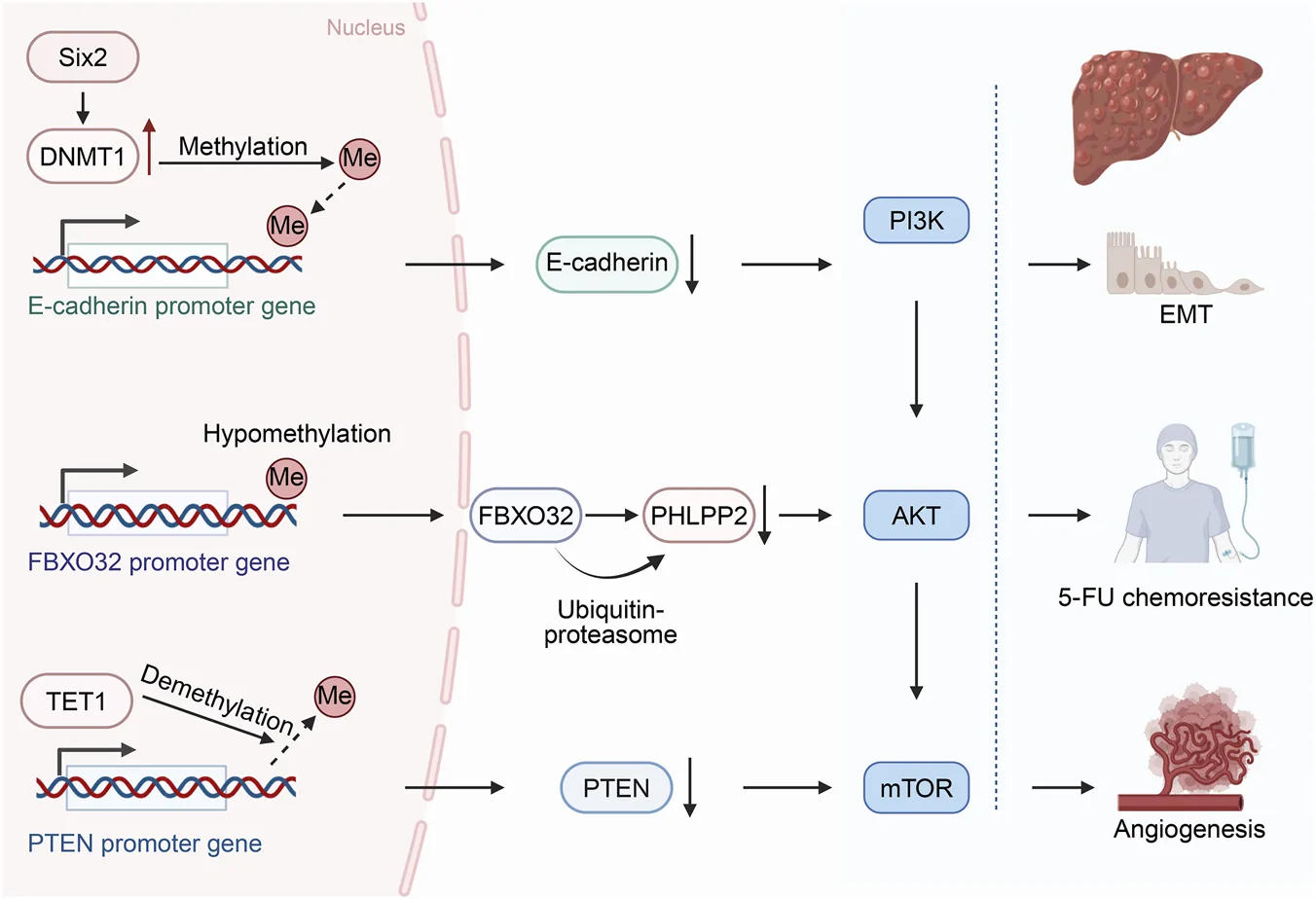
DNA methylation-dependent regulation of PI3K/AKT signaling in HCC. The schematic shows how disrupted DNA methylation can alter PI3K/AKT signaling and promote EMT, 5-FU resistance, and angiogenesis. Figure created with BioRender.com. HCC, hepatocellular carcinoma; EMT, epithelial-mesenchymal transition; me, methylation; Six2, SIX homeobox 2; DNMT1, DNA methyltransferase 1; TET1, tet methylcytosine dioxygenase 1; FBXO32, F-box protein 32; PHLPP2, PH domain leucine-rich repeat protein phosphatase 2; PTEN, phosphatase and tensin homolog; E-cadherin, epithelial cadherin; mTOR, mechanistic target of rapamycin; 5-FU, 5-fluorouracil.

Collectively, these findings demonstrate that aberrant DNA methylation is a key upstream driver of sustained PI3K/AKT signaling. Altered methylation status causes a loss of tumor-suppressive restraints and an increase in oncogenic transcription, which together promote HCC initiation and progression.

### 4.2 RNA-mediated post-transcriptional and protein-level regulation of PI3K/AKT signaling

Non-coding RNAs regulate PI3K/AKT pathway activity and contribute to malignant progression in HCC (Hussain et al., 2024). lncRNAs can activate the pathway by altering RNA metabolism or protein stability. IGFRIL is an oncogenic lncRNA that regulates PTBP1-dependent RNA metabolism and is upregulated in HCC tissues. IGFRIL recruits PTBP1 and promotes IGFBP3 mRNA degradation, thereby reducing IGFBP3 protein abundance. The resulting activation of the IGF1R/AKT/mTOR axis promotes HCC progression (Zhang et al., 2025a). The transcription factor SP1 also binds the USP27X-AS1 promoter and increases its transcription. USP27X-AS1 recruits the deubiquitinase USP7, which limits AKT ubiquitination and degradation. Increased AKT stability then sustains PI3K/AKT signaling and promotes HCC cell proliferation and metastasis (Su C. et al., 2024).

Several hypoxia-responsive lncRNAs regulate AKT signaling through feedback loops. Hypoxia-inducible factor-1α (HIF-1α) induces lncRNA KDM4A-AS1. KDM4A-AS1 sponges miR-411-5p, increases KPNA2 expression, and activates AKT signaling. These interactions establish a KDM4A-AS1/KPNA2/HIF-1α positive feedback loop that amplifies oncogenic signaling (Chen et al., 2021). The transcription factor E2F1 can also upregulate KDM4A-AS1. The lncRNA then recruits ILF3 to stabilize AURKA mRNA, increasing AURKA expression and PI3K/AKT pathway activity. This mechanism promotes HCC cell proliferation, invasion, and EMT (Shen et al., 2023). HIF-1α also induces lncRNA MAPKAPK5-AS1, which sponges miR-154-5p and increases PLAGL2 expression. PLAGL2 activates PI3K/AKT signaling and, in turn, increases HIF-1α expression, establishing another positive feedback loop (Wang et al., 2021). The lncRNA RP11-295G20.2 is upregulated in HCC and binds PTEN. It promotes lysosomal degradation of PTEN, relieving PTEN-mediated inhibition of AKT and activating PI3K/AKT signaling. RP11-295G20.2 also suppresses autophagy, which may further support HCC progression (Liang et al., 2021).

Circular RNAs (circRNAs) commonly regulate PI3K/AKT signaling through competing endogenous RNA (ceRNA) mechanisms. circMRPS35 sponges miR-148a and relieves repression of the downstream target STX3. Increased STX3 promotes PTEN ubiquitination and degradation, thereby weakening PTEN-mediated pathway inhibition and sustaining PI3K/AKT activation (Li et al., 2022). circACVR2A similarly binds and inhibits tumor-suppressive miR-511-5p. This interaction activates PI3K/AKT signaling and enhances HCC cell proliferation, invasion, and resistance to apoptosis (Fei et al., 2024). By contrast, circZKSCAN1 can encode the functional polypeptide circZKSaa. circZKSaa promotes FBXW7-mediated ubiquitination and degradation of mTOR, restraining PI3K/AKT/mTOR signaling. This activity suppresses HCC progression and increases sensitivity to sorafenib (Figure 3; Song et al., 2023).

**FIGURE 3:**
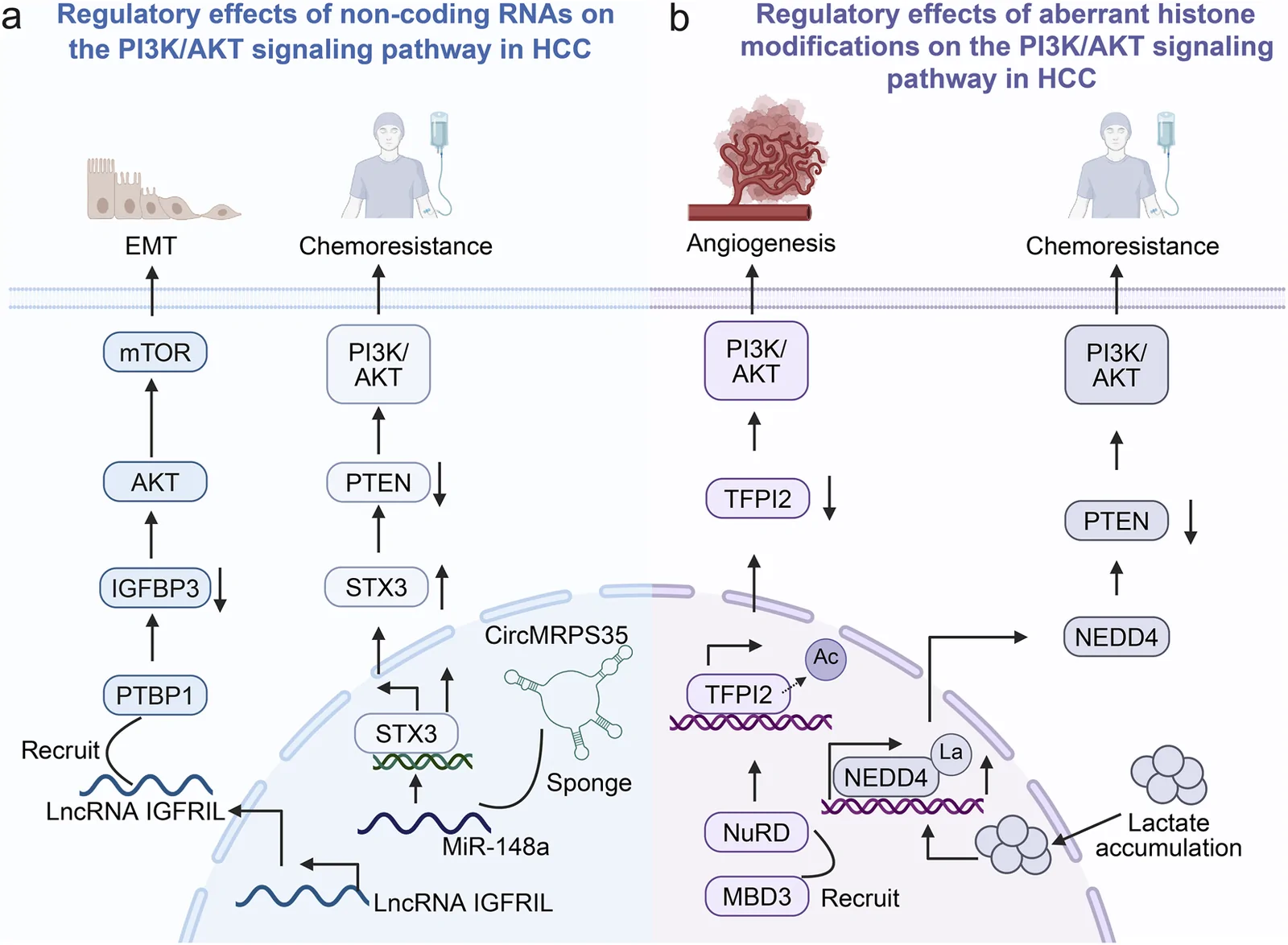
Non-coding RNA- and histone modification-dependent regulation of PI3K/AKT signaling in HCC. **(a)** The left panel shows representative non-coding RNA-mediated PI3K/AKT regulation in HCC, depicting EMT and chemoresistance. **(b)** The right panel illustrates aberrant histone modification-dependent PI3K/AKT modulation, including angiogenesis and chemoresistance. Figure created with BioRender.com. HCC, hepatocellular carcinoma; EMT, epithelial-mesenchymal transition; lncRNA, long non-coding RNA; circRNA, circular RNA; miR, microRNA; PTEN, phosphatase and tensin homolog; STX3, syntaxin 3; IGFBP3, insulin-like growth factor-binding protein 3; PTBP1, polypyrimidine tract-binding protein 1; mTOR, mechanistic target of rapamycin; TFPI2, tissue factor pathway inhibitor 2; NEDD4, neural precursor cell expressed developmentally downregulated protein 4; MBD3, methyl-CpG-binding domain protein 3; NuRD, nucleosome remodeling and deacetylase complex; la, lactylation; ac, acetylation.

### 4.3 Effects of aberrant histone modifications on PI3K/AKT signaling

Histone modifications also influence HCC progression by regulating PI3K/AKT pathway activity (Bueloni et al., 2025). Histone-modifying enzymes, including writers and erasers, establish or remove specific histone marks. Reader proteins recognize these marks and recruit transcriptional regulatory or chromatin-remodeling complexes, thereby converting epigenetic signals into transcriptional responses (Yang and Wu, 2025; Kawaf et al., 2024; Wang et al., 2026). Consequently, aberrant histone modifications and dysregulated reader-protein activity may alter PI3K/AKT regulator expression, AKT phosphorylation, and negative-feedback mechanisms. These effects may ultimately sustain PI3K/AKT activation in HCC.

Altered histone methylation can activate the pathway by derepressing oncogenes or changing the expression of pathway components. Reduced PCK1 expression in hepatocytes lowers intracellular S-adenosylmethionine (SAM) synthesis and decreases H3K9me3. The resulting derepression of S100A11 increases its expression. S100A11 binds and activates AKT1, sustaining PI3K/AKT signaling and promoting HCC cell proliferation and invasion (Gou et al., 2023). Overexpressed EZH2 also deposits H3K27me3 at the miR-622 promoter and silences miR-622, thereby relieving repression of CXCR4. Binding of CXCL12 to CXCR4 activates both PI3K/AKT and RAF/MEK/ERK signaling, which together promote HCC progression (Wang et al., 2023).

Altered histone acetylation can regulate PI3K/AKT signaling through mechanisms in both the tumor microenvironment and tumor cells. In tumor-associated macrophages (TAMs), GOLM1 binds ACLY and blocks PKA-mediated ACLY phosphorylation. This interaction lowers nuclear ACLY activity and acetyl-CoA production, reducing the activating H3K27ac mark at the EFEMP1 locus. Reduced EFEMP1 transcription and secretion limit its binding to EGFR on HCC cells. This restrains PI3K/AKT and MAPK signaling, suppressing malignant progression (Zhen et al., 2026). In HCC cells, MBD3 recruits the NuRD complex to deacetylate histones at the TFPI2 promoter and silence TFPI2 transcription. Because TFPI2 negatively regulates PI3K/AKT signaling, its loss permits sustained pathway activation. This mechanism promotes HCC cell proliferation, invasion, metastasis, and tumor angiogenesis (Yan et al., 2022).

Histone lactylation links glucose metabolic reprogramming to PI3K/AKT pathway activation in HCC. PYCR1 overexpression increases glycolysis and intracellular lactate accumulation. Lactate promotes H3K18 lactylation at the IRS1 promoter and increases IRS1 transcription. Increased IRS1 expression subsequently activates PI3K/AKT/mTOR and MAPK/ERK signaling, promoting HCC cell proliferation and metastasis (Wang et al., 2024). Lactate-dependent histone modification may also contribute to chemoresistance. Glycolysis-driven lactate accumulation increases H3K14 lactylation and NEDD4 expression. NEDD4 then promotes PTEN ubiquitination and degradation, relieving PTEN-mediated inhibition of PI3K/AKT/mTOR signaling and supporting chemoresistance (Zeng et al., 2025).

## 5 PI3K/AKT-mediated epigenetic modifications in HCC

The preceding sections describe several mechanisms through which epigenetic alterations sustain PI3K/AKT activation and promote HCC progression. Evidence for the reverse relationship, in which activated PI3K/AKT signaling modifies the epigenetic state of HCC cells, is more limited. Consequently, the bidirectional regulatory network remains incomplete. The mechanisms reported to date are summarized below (Figure 4).

**FIGURE 4:**
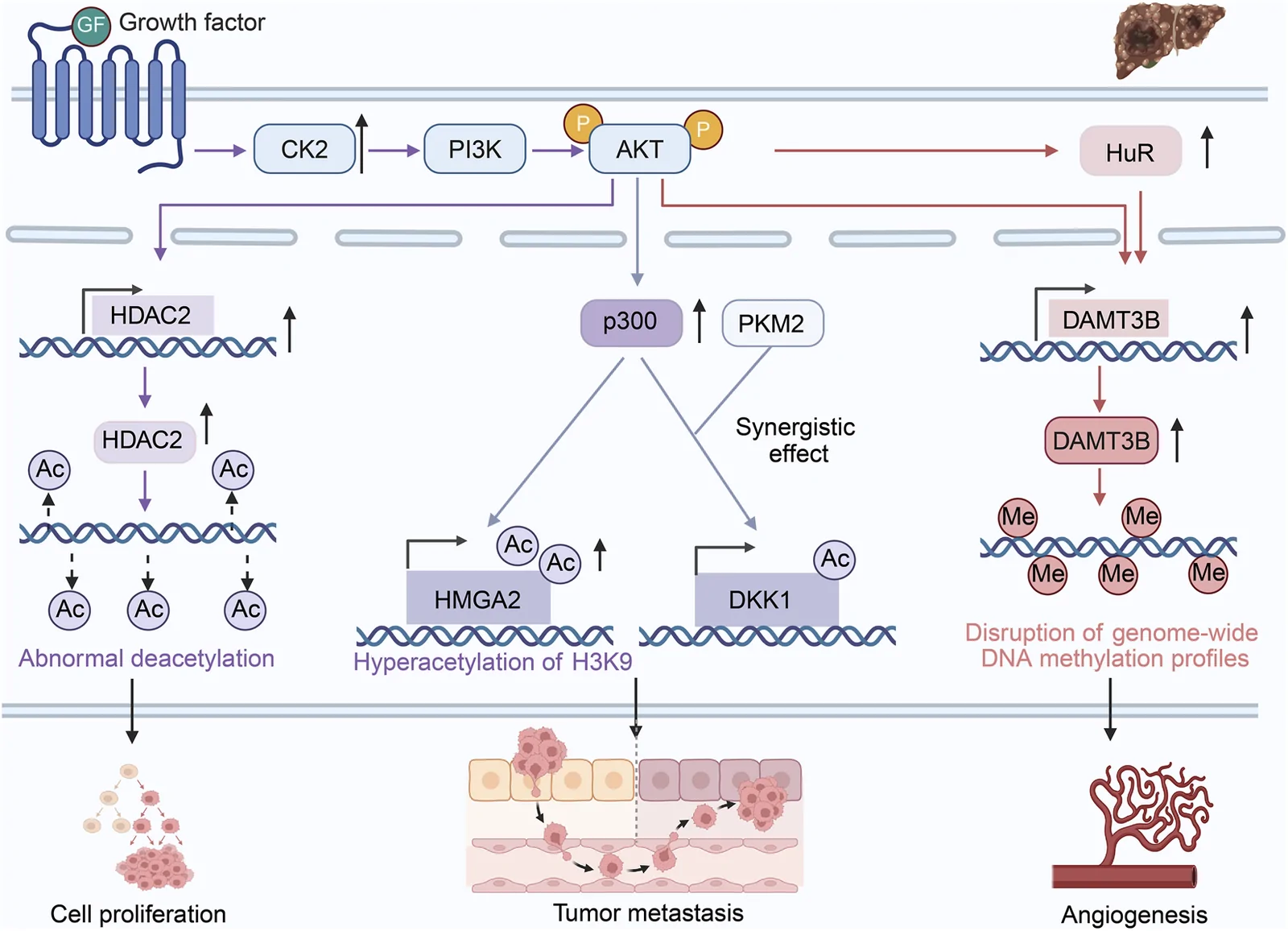
PI3K/AKT-mediated epigenetic remodeling in HCC. The schematic shows mechanisms through which PI3K/AKT signaling alters epigenetic regulation and promotes EMT, cell proliferation, and angiogenesis. Figure created with BioRender.com. HCC, hepatocellular carcinoma; EMT, epithelial-mesenchymal transition; HDAC2, histone deacetylase 2; p300, E1A-binding protein p300; PKM2, pyruvate kinase M2; HuR, human antigen R; DNMT3B, DNA methyltransferase 3B; HMGA2, high mobility group AT-hook 2; DKK1, Dickkopf WNT signaling pathway inhibitor 1; ac, acetylation; me, methylation.

Activated AKT binds the DNMT3B promoter and increases DNMT3B transcription. AKT also upregulates the RNA-binding protein HuR, which extends DNMT3B mRNA half-life and increases DNMT3B translation. These mechanisms raise DNMT3B protein abundance, alter genome-wide DNA methylation, and promote malignant HCC phenotypes (Mei et al., 2010). AKT also regulates the acetyltransferase p300. Phosphorylation of p300 at Ser1834 enhances its acetyltransferase activity and increases H3K9 acetylation at the HMGA2 promoter. The resulting increase in HMGA2 expression promotes HCC cell invasion and metastasis (Liang et al., 2021). Activated AKT also acts through p300 to increase H3K9 acetylation at the DKK1 promoter. At the same locus, PKM2, acting downstream of MEK/ERK, mediates H3T11 phosphorylation. Together, these modifications increase DKK1 expression and the invasive and metastatic potential of HCC cells (Niu et al., 2020). Other studies indicate that EGF stimulation increases CK2α expression and activates PI3K/AKT signaling, leading to higher histone deacetylase 2 (HDAC2) expression. HDAC2 then increases the abundance of cell-cycle proteins, including Cdc25c and cyclin B, accelerates the G2/M transition, and promotes HCC cell proliferation (Kim et al., 2014).

Taken together, the reviewed evidence indicates that epigenetic alterations and PI3K/AKT signaling form a bidirectional regulatory network in HCC. This network depends on the underlying hepatic disease context and contributes to tumor development and progression. HCC commonly arises in the setting of chronic liver injury, persistent inflammation, liver fibrosis or cirrhosis, metabolic dysregulation, and hypoxia-associated immune-stromal remodeling. These pathological conditions may reshape DNA methylation, non-coding RNA, and histone-modification patterns, thereby influencing PI3K/AKT pathway activity. Conversely, sustained PI3K/AKT activation may further reinforce malignant transformation, metabolic reprogramming, and therapeutic resistance by modulating factors that regulate chromatin and epigenetic states. This epigenetic-PI3K/AKT regulatory network may vary according to HCC etiology and the pathological state of the liver. HBV-associated HCC is characterized by viral DNA integration and HBx-related transcriptional and epigenetic dysregulation, whereas HCV-associated HCC is driven mainly by chronic inflammatory liver injury. Accordingly, HBV- and HCV-associated HCCs display distinct DNA methylation and transcriptional profiles (Huang et al., 2025; Low et al., 2022; Aydin et al., 2025). Metabolic dysfunction-associated steatotic liver disease (MASLD)-associated HCC exhibits close crosstalk between metabolic reprogramming and epigenetic alterations, driven by insulin resistance, lipotoxicity, oxidative stress, and chronic metabolic inflammation (Li A. et al., 2024; Aiello et al., 2025). HCC arising in cirrhotic and non-cirrhotic livers also differs in its tumorigenic context and clinical presentation. Notably, a substantial proportion of MASLD-associated HCC develops without cirrhosis and may present with larger or more advanced tumors (Karaoğullarından et al., 2023; Mi et al., 2025). These differences may influence PI3K/AKT pathway activity and tumor sensitivity to epigenetic or PI3K/AKT-directed therapies. However, direct comparative studies across etiological and cirrhosis-defined HCC subgroups remain limited.

## 6 Targeting PI3K/AKT signaling and epigenetic regulation in HCC

This section reviews agents that target PI3K/AKT signaling or epigenetic regulators in HCC, with an emphasis on target selectivity, clinical development, toxicity, and limitations. It also considers the rationale and the preclinical evidence for combining PI3K/AKT pathway inhibitors with epigenetic-targeting agents (Table 2).

**TABLE 2:** Selected clinical studies of PI3K/AKT-, epigenetic-, and RNA-targeting agents relevant to HCC.

Agent class	Agents	Cancer type	Trial identifier	Phase	Status	Country	Date first posted
PI3K inhibitors	Buparlisib	Solid tumors	NCT02439489	Phase Ib	Completed	Italy	2012–12
	Copanlisib	Hepatocellular carcinoma	NCT03735628	Phase 1	Completed	United States	2018–10–17
AKT inhibitors	MK-2206	Adult hepatocellular carcinoma	NCT01239355	Phase 2	Terminated	United States	2010–12
	Capivasertib	Solid tumor	NCT04958226	Phase 1	Completed	United States	2021–10–15
	Ipatasertib	Neoplasms	NCT01562275	Phase 1	Completed	United States, Spain	2012–04
	Miransertib	Malignant lymphoma Solid tumor, tumor	NCT01473095	Phase 1	Completed	United States	2011–11
Dual PI3K/mTOR inhibitors	Dactolisib	Malignant solid tumor	NCT01343498	Phase 1	Completed	United States	2011–04
	Samotolisib	Advanced cancer	NCT01655225	Phase 1	Completed	United States	2012–07–31
DNA methyltransferase inhibitors	Guadecitabine SGI-110	Hepatocellular carcinoma	NCT01752933	Phase 2	Completed	United States	2012–12
Histone modification-targeting agents	Resminostat	Hepatocellular carcinoma	NCT00943449	Phase 2	Completed	Germany	2009–07
	Belinostat	Adult primary hepatocellular carcinoma Advanced adult primary liver cancer	NCT00321594	Phase 1/Phase 2	Completed	United States Singapore	2006–05
	Vorinostat	Tumors	NCT00127127	Phase 1	Completed	Location not provided	2005–06–10
Chromatin remodeling inhibitors	CBL0137	Solid tumors	NCT01905228	​	Completed	United States	2013–07
Non-coding RNA-targeting agents	MRX34	Renal cell carcinoma	NCT01829971	Phase 1	Terminated	United States	2013–04
	TKM-080301	Hepatocellular carcinoma Hepatoma Liver cancer, adult Liver cell carcinoma, adult	NCT02191878	Phase 1/Phase 2	Completed	United States	2014–06
	LNA-i-miR-221	Advanced solid tumor Hepatocarcinoma	NCT04811898	Phase 1	Completed	Italy	2019–01–14

### 6.1 Targeting the PI3K/AKT signaling pathway

PI3K/AKT pathway inhibitors are classified according to target selectivity, but their clinical value in HCC remains uncertain (Schmitz et al., 2025; Wang, 2025). The oral pan-class I PI3K inhibitor buparlisib showed poor tolerability in a multicenter phase II basket trial that included a small cohort of patients with advanced HCC. Adverse events included elevated transaminases, anxiety, and depression, which limited sustained treatment (Piha-Paul et al., 2019; Wu et al., 2016). By contrast, the 4-aminoquinazoline compound DHW-208 has been evaluated only in cell and animal models. In those studies, it showed limited toxicity to normal hepatocytes and major organs at therapeutically active doses, but no human trial has been reported (Wang et al., 2022).

Idelalisib is a selective PI3Kδ inhibitor approved for hematologic malignancies, but it has not been evaluated in HCC trials. Its suppressive effects on cytotoxic T cells and natural killer cells may further limit its use in patients with advanced HCC and impaired immunity (Ko et al., 2018; Lee et al., 2020).

Dual PI3K/mTOR inhibitors target both PI3K and mTORC1/2, thereby blocking multiple nodes in the PI3K/AKT/mTOR cascade (Wu et al., 2022). Dactolisib (BEZ235) has undergone exploratory phase I and II trials in patients with advanced solid tumors, but these studies did not include dedicated advanced HCC cohorts. Severe diarrhea was the principal dose-limiting toxicity, and liver function abnormalities also occurred. Further development was suspended because of limited antitumor activity and poor long-term tolerability (Masuda et al., 2011; Chang et al., 2013). Samotolisib (LY3023414), another dual inhibitor, was tested in multicenter phase I trials in solid tumors. These trials included only a small number of patients with advanced HCC. Disease control was transient in a subset of patients. Hyperglycemia, elevated transaminases, and other treatment-related toxicities frequently limited long-term treatment (Smith et al., 2016; Bendell et al., 2018; Kondo et al., 2020).

Small-molecule AKT inhibitors include allosteric and ATP-competitive agents. Both classes have reached phase I evaluation in patients with advanced solid tumors (Mroweh et al., 2021; Pervanidis et al., 2024). The allosteric pan-AKT inhibitor MK-2206 has been studied mainly in phase I trials. Reported toxicities include rash, nausea, pruritus, hyperglycemia, and diarrhea, and dedicated phase II data for HCC are unavailable (Yap et al., 2014; Yap et al., 2011). Capivasertib is an ATP-competitive pan-AKT inhibitor with manageable pharmacokinetics and an acceptable safety profile in phase I solid-tumor studies. Its most frequent adverse events were hyperglycemia, diarrhea, and rash. Dedicated trials in HCC have not yet been initiated (Zhang et al., 2025b; Fernandez-Teruel et al., 2024).

### 6.2 Targeting epigenetic regulation

Small molecules targeting enzymes that mediate epigenetic modifications may reverse therapeutic resistance or alter the tumor microenvironment. However, their clinical value in advanced HCC remains under investigation (Fernández-Barrena et al., 2020; Oura et al., 2023).

Decitabine and azacitidine are nucleoside analogs that inhibit DNA methyltransferases. Monotherapy studies in solid tumors have largely remained in phase I, and few combination regimens have progressed to early phase II trials. Myelosuppression is a dose-limiting toxicity of both drugs. Patients with liver metastases or preexisting hepatic dysfunction may also be susceptible to mild-to-moderate drug-induced liver injury (Ye et al., 2024; Hu et al., 2021; Vigil et al., 2010; Mei et al., 2015).

Vorinostat and resminostat are HDAC inhibitors with different subtype selectivities, which may contribute to their distinct safety profiles when combined with sorafenib (Tsilimigras et al., 2018). A phase I trial of the broad-spectrum pan-HDAC inhibitor vorinostat plus sorafenib has been completed. Thrombocytopenia and liver injury were frequent dose-limiting toxicities, and many participants required dose modification. No recommended phase II dose was established (Gordon et al., 2019). By contrast, the subtype-selective inhibitor resminostat was evaluated in the phase I/II second-line SHELTER trial, in which mild gastrointestinal adverse events predominated. Although cross-trial comparisons require caution, the available data suggest better overall tolerability for resminostat plus sorafenib than for the vorinostat combination (Bitzer et al., 2016; Hao et al., 2025).

Chromatin-directed agents include BRD9 and FACT complex inhibitors (Nagaraju et al., 2022). The selective BRD9 inhibitor BI-9564 has been evaluated only in preclinical cell and animal models, and clinical data in HCC are unavailable (Wei et al., 2026). The FACT inhibitor CBL0137 has been tested in a phase I trial in patients with advanced solid tumors. That study included only a small number of patients with HCC and no dedicated HCC cohort. The available data are therefore insufficient to determine efficacy in this disease (Shen et al., 2020).

Non-coding RNA-targeted agents investigated in HCC include the siRNA agent TKM-080301 (TKM-PLK1) and the LNA anti-miR inhibitor LNA-i-miR-221 (Grillone et al., 2024). TKM-080301 was evaluated in a multicenter phase I trial involving 43 patients with Child-Pugh A HCC. Grade 4 thrombocytopenia was the dose-limiting toxicity, and antitumor activity was modest, limiting further development of the agent as monotherapy (El et al., 2019). LNA-i-miR-221 was administered to only one patient with HCC in a phase I pan-solid-tumor study. No grade 3 or 4 adverse events occurred in any dose group, but the single HCC case precludes conclusions about disease-specific safety or efficacy (Tassone et al., 2023).

### 6.3 Combining PI3K/AKT inhibitors with epigenetic-targeting agents

In cell and animal models of HCC, the HDAC inhibitor vorinostat reduces AKT phosphorylation at Ser473 and enhances the cytotoxicity of targeted agents. These findings provide a mechanistic rationale for combining HDAC inhibitors with AKT inhibitors such as MK-2206 or capivasertib (Yan et al., 2024). Dual PI3K/HDAC compounds offer another approach. CUDC-907 and BEBT-908 each suppress kinase signaling and epigenetic regulation within a single molecule. They show greater preclinical antitumor activity than the corresponding single-agent combinations (Lima and Lima, 2026). DNMT inhibitors can also restore PTEN expression and reduce AKT activation, supporting their combination with AKT inhibitors (Mohite and Doshi, 2024). Nevertheless, no regimen combining pathway-directed and epigenetic therapy is approved for HCC. Additional *in vivo* and clinical evidence is needed before these preclinical findings can guide treatment.

## 7 Challenges and prospects

Epigenetic regulation of PI3K/AKT signaling is well documented, whereas evidence for PI3K/AKT-driven epigenetic reprogramming remains limited (Wan et al., 2026; Wali et al., 2025; Zhou et al., 2024). The full bidirectional network therefore requires further validation. Sufficiently specific and sensitive biomarkers for early HCC detection are still lacking in clinical practice. The limited sensitivity of serum alpha-fetoprotein contributes to late diagnosis, when curative interventions are often no longer feasible (Hanif et al., 2022; Rogers et al., 2026; Oussalah et al., 2026; Park et al., 2025). Most PI3K/AKT pathway inhibitors and epigenetic agents remain in preclinical development or early phase I/II trials for HCC (Zheng et al., 2025; Cai et al., 2026). Idelalisib is approved for hematologic malignancies but not for HCC (Banerjee et al., 2023; Skånland et al., 2024). Dose-limiting hyperglycemia, hepatic injury, and adaptive resistance can limit prolonged treatment (Sabbah et al., 2024). Variable dosing schedules, treatment sequences, and HCC molecular heterogeneity further complicate response assessment. They also limit the clinical utility of current monotherapies and simple combinations (Yang et al., 2024; Müller et al., 2025; Safri et al., 2024).

Future research should focus on patient selection and biomarker development for PI3K/AKT-epigenetic combination therapy in HCC (Ghosh et al., 2025; Rajak et al., 2025). Multi-omics analyses may help establish composite biomarkers that integrate PI3K/AKT pathway activity, epigenetic alterations, genetic background, and liver function. These biomarkers could identify patients most likely to benefit and predict treatment response (Lu M. Y. et al., 2026). HCC-specific trial cohorts are needed to distinguish disease-specific activity and toxicity from findings in pan-solid-tumor studies (Chew et al., 2024; Chodup et al., 2025; Finn et al., 2025). Future studies should define the optimal sequence of PI3K/AKT-epigenetic therapy, including upfront combination, monotherapy, or sequential treatment after resistance. Biomarker-guided trials should also establish the recommended phase II dose, characterize dose-limiting toxicities, and define dose-adjustment strategies based on hepatic reserve, pharmacokinetic exposure, and treatment-related adverse events (Li et al., 2026; Qi et al., 2025; Liao et al., 2024). Patients receiving combination regimens should be carefully monitored for overlapping toxicities, including hyperglycemia, hepatic injury, and cytopenias. Treatment discontinuation due to toxicity should also be monitored (Moris et al., 2025; Gujarathi et al., 2024). Evidence from established immunotherapy combinations in HCC supports the importance of rational combination design (Liu et al., 2023c; Bloom et al., 2025). Although these immunotherapy combinations demonstrate clinical benefits, they do not validate the addition of PI3K/AKT or epigenetic agents. Liver dysfunction and tumor heterogeneity may also influence treatment response and resistance. Future studies should therefore incorporate hepatic reserve and liver function into patient stratification and safety monitoring. Longitudinal sampling should identify responsive subgroups, monitor tumor evolution, and elucidate treatment resistance mechanisms (Zhao S. et al., 2025; Lv et al., 2026). Imaging, artificial intelligence, and nanocarrier systems may complement diagnosis, response prediction, treatment monitoring, and precision drug delivery. Their clinical utility requires further validation in large-scale prospective studies (Huang et al., 2026; Lu T. et al., 2026; Li Z. et al., 2024; Peng et al., 2026; Jia et al., 2026). Overall, priorities include patient selection, biomarker development, treatment sequence and dosage, combination-related toxicity, and the effects of liver function and tumor heterogeneity on outcomes.

## 8 Conclusion

Dysregulated DNA methylation, non-coding RNAs, and histone modifications can sustain PI3K/AKT pathway activation and promote HCC cell proliferation, invasion, and therapeutic resistance. Evidence that PI3K/AKT signaling reshapes the epigenome is more limited, leaving aspects of this bidirectional relationship unresolved. Most PI3K/AKT inhibitors and DNMT- or HDAC-targeting agents remain in preclinical development or early-phase trials for HCC. No standard monotherapy or combination regimen involving these agents has been approved. Dose-limiting toxicity, adaptive resistance, inconsistent treatment schedules, and molecular heterogeneity remain major barriers. Preclinical studies support combined pathway and epigenetic inhibition, but the available evidence is insufficient for clinical adoption. Priorities include clarifying how PI3K/AKT signaling reshapes the epigenome, validating epigenetic biomarkers, and testing rational combinations in adequately powered HCC-specific trials. Nanocarrier-based co-delivery may improve the therapeutic index, but it also requires rigorous *in vivo* and clinical validation.
